# Length of Initial Prescription at Hospital Discharge and Long-Term Medication Adherence for Elderly, Post-Myocardial Infarction Patients: Protocol for an Interrupted Time Series Study

**DOI:** 10.2196/18981

**Published:** 2020-11-04

**Authors:** J D Schwalm, Noah M Ivers, Zachary Bouck, Monica Taljaard, Madhu K Natarajan, Lisa Dolovich, Kednapa Thavorn, Tara McCready, Erin O'Brien, Jeremy M Grimshaw

**Affiliations:** 1 Population Health Research Institute McMaster University and Hamilton Health Sciences Hamilton, ON Canada; 2 Division of Cardiology Department of Medicine McMaster University Hamilton, ON Canada; 3 Family Practice Health Centre Women's College Hospital Toronto, ON Canada; 4 Department of Family and Community Medicine University of Toronto Toronto, ON Canada; 5 ICES Toronto, ON Canada; 6 Dalla Lana School of Public Health University of Toronto Toronto, ON Canada; 7 Centre for Drug Policy and Evaluation Unity Health Toronto Toronto, ON Canada; 8 Clinical Epidemiology Program Ottawa Hospital Research Institute University of Ottawa Ottawa, ON Canada; 9 School of Epidemiology and Public Health University of Ottawa Toronto, ON Canada; 10 Leslie Dan Faculty of Pharmacy University of Toronto Toronto, ON Canada; 11 Department of Medicine University of Ottawa Ottawa, ON Canada

**Keywords:** post-myocardial infarction, adherence, standardized discharge prescription form, secondary prevention, policy change, medication, elderly, intervention, prescription, discharge, prevention, cardiology, heart

## Abstract

**Background:**

Based on high-quality evidence, guidelines recommend the long-term use of secondary prevention medications post-myocardial infarction (MI) to avoid recurrent cardiovascular events and death. Unfortunately, discontinuation of recommended medications post-MI is common. Observational evidence suggests that prescriptions covering a longer duration at discharge from hospital are associated with greater long-term medication adherence. The following is a proposal for the first interventional study to evaluate the impact of longer prescription duration at discharge post-MI on long-term medication adherence.

**Objective:**

The overarching goal of this study is to reduce morbidity and mortality among post-MI patients through improved long-term cardiac medication adherence. The specific objectives include the following. First, we will assess whether long-term cardiac medication adherence improves among elderly, post-MI patients following the implementation of (1) standardized discharge prescription forms with 90-day prescriptions and 3 repeats for recommended cardiac medication classes, in combination with education and (2) education alone compared to (3) usual care. Second, we will assess the cost implications of prolonged initial discharge prescriptions compared with usual care. Third, we will compare clinical outcomes between longer (>60 days) versus shorter prescription durations. Fourth, we will collect baseline information to inform a multicenter interventional study.

**Methods:**

We will conduct a quasiexperimental, interrupted time series design to evaluate the impact of a multifaceted intervention to implement longer duration prescriptions versus usual care on long-term cardiac medication adherence among post-MI patients. Intervention groups and their corresponding settings include: (1) intervention group 1: 1 cardiac center and 1 noncardiac hospital allocated to receive standardized discharge prescription forms supporting the dispensation of 90 days’ worth of cardiac medications with 3 repeats, coupled with education; (2) intervention group 2: 4 sites (including 1 cardiac center) allocated to receive education only; and (3) control group: all remaining hospitals within the province that did not receive an intervention (ie, usual care). Administrative databases will be used to measure all outcomes. Adherence to 4 classes of cardiac medications — statins, beta blockers, angiotensin system inhibitors, and secondary antiplatelets (ie, prasugrel, clopidogrel, or ticagrelor) — will be assessed.

**Results:**

Enrollment began in September 2017, and results are expected to be analyzed in late 2020.

**Conclusions:**

The results have the potential to redefine best practices regarding discharge prescribing policies for patients post-MI. A policy of standardized maximum-duration prescriptions at the time of discharge post-MI is a simple intervention that has the potential to significantly improve long-term medication adherence, thus decreasing cardiac morbidity and mortality. If effective, this low-cost intervention to implement longer duration prescriptions post-MI could be easily scaled.

**Trial Registration:**

ClinicalTrials.gov NCT03257579; https://clinicaltrials.gov/ct2/show/NCT03257579

**International Registered Report Identifier (IRRID):**

DERR1-10.2196/18981

## Introduction

### Background

**Figure 1:**
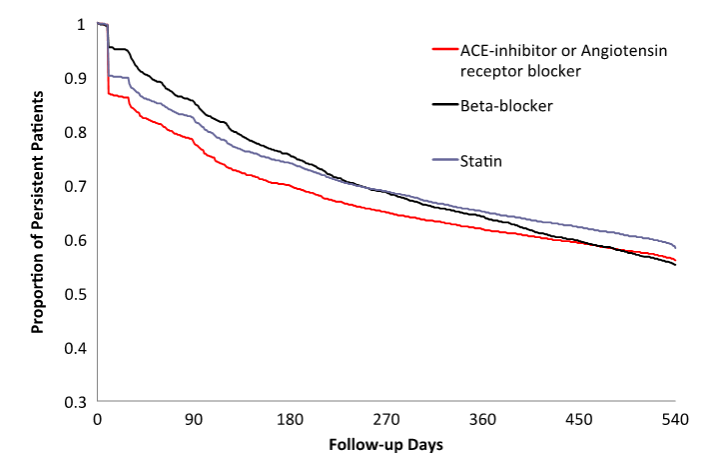
Persistence with secondary prevention medications after angiography. ACE: angiotensin-converting enzyme.

International guidelines recommend the long-term use of secondary preventative cardiac medications following a myocardial infarction (MI) [1-3]. The medication classes used for secondary prevention post-MI include both a primary antiplatelet (aspirin) and secondary antiplatelet (eg, prasugrel, clopidogrel, or ticagrelor), statins, beta blockers, and angiotensin system inhibitors (ie, an angiotensin-converting enzyme inhibitor or angiotensin receptor blocker). These medications provide an expected 60% relative risk reduction of recurrent cardiovascular events [1,2]. However, studies have shown that postdischarge cardiac medication adherence tends to steadily decline over time (Figure 1) [4-12]. Multiple studies have shown an increased risk of morbidity and mortality associated with medication nonadherence in patients with coronary artery disease (CAD) [10-14].

Although medication adherence is a complex phenomenon with multiple interacting factors, there is emerging evidence that a simple step taken by health care providers at the time of hospital discharge plays an important role in long-term adherence. In a population-based observational study of over 18,000 patients conducted by our research team, greater long-term adherence to cardiac medications was observed in Ontario patients receiving longer initial prescriptions (>60 days versus <31 days) at the time of discharge [4]. Specifically, a higher proportion of patients receiving longer duration prescriptions had greater than 80% proportion of days covered (PDC) in the 18 months following hospital discharge for angiotensin system inhibitors (adjusted odds ratio [OR] 4.1, 95% CI 3.6-4.7), beta blockers (adjusted OR 2.4, 95% CI 1.9-3.1), and statins (adjusted OR 3.0, 95% CI 2.6-3.4) [4]. Two earlier studies found similar results. Batal et al [15] analyzed a cohort of 3386 patients from a large US health care center and found higher adherence to statins in patients with mostly 60-day prescriptions as compared to those with mostly 30-day prescriptions (adjusted risk ratio 1.4, 95% CI 1.3-1.6). Steiner et al [16] examined maintenance of medication in 290 outpatients from Veteran Affairs Centers in the United States and found that those with longer prescriptions for digoxin (ie, at least a 90-day supply) were more likely to regularly acquire the medication over 9 to 14 months. While these 3 observational studies suggest an association between prescription duration and long-term medication adherence, evidence from a prospective study is required to support implementation of this policy as a best practice.

### Rationale and Study Objectives

Poor medication adherence was identified by the World Health Organization in 2003 as a worldwide challenge in the management of chronic diseases [17]. The consequences of suboptimal adherence include undesirable health outcomes as well as increased health care costs. Both provider-level and system-level factors play important roles in optimizing adherence. We have previously shown that inappropriate beliefs regarding risk perception and outcome expectancies, prescription refill burden, and unintentional forgetfulness due to lack of habituation all contribute to the observed general decline in medication adherence over time [18-20]. Providing longer prescriptions at the time of discharge can address all of these barriers. However, prescriptions covering ≥90 days are currently provided to only 21% of all cardiac patients and to no more than 54% of MI patients, suggesting an opportunity for this change to benefit many patients [4,21].

We know of no guidelines or practice standards for physicians that recommend the prescription of maximum duration (ie, ≥90 days) for cardiac medications at discharge. The use of standardized hospital-based medication discharge practices has been shown to minimize medication errors and to optimize guideline-recommended therapies [22]. However, no prospective study has evaluated the effects of implementing an institutional policy or standardized discharge cardiac medication prescriptions of maximum duration on long-term medication adherence. Therefore, it is hypothesized that longer prescription duration at discharge will promote better long-term cardiac medication adherence.

The specific objectives of this study include estimating and comparing the effects on long-term cardiac medication adherence of (1) a standardized discharge form featuring prescription duration of 90 days with 3 repeats in post-MI patients in combination with education, (2) education only, and (3) usual care (ie, no intervention regarding prescription duration) and assessing the economic implications of the prolonged initial discharge prescriptions as compared to usual care. To our knowledge, this project is the first interventional study to investigate how modifying prescription duration impacts medication adherence.

## Methods

### Trial Setting and Design

The Myocardial Infarction Prescription Adherence Duration (MIPAD) study will use a quasiexperimental, interrupted time series design to evaluate the impact of standardized prolonged discharge prescription forms and education versus usual care on long-term cardiac medication adherence among elderly, post-MI patients in Ontario who were discharged from hospital following a cardiac catheterization procedure between September 1, 2015 and August 31, 2018.

**Figure 2:**
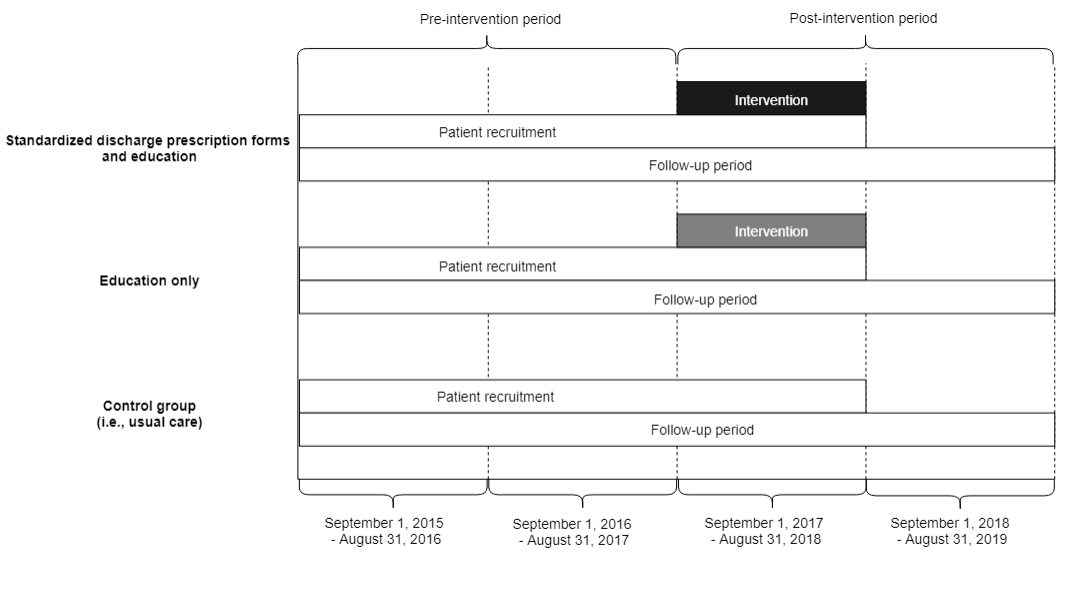
Intervention and control groups in the Myocardial Infarction Prescription Adherence Duration (MIPAD) study.

After the first 24 months within the study window (ie, the pre-intervention period: September 1, 2015 through August 31, 2017), a total of 6 sites (2 of which are cardiac centers, where advanced cardiac procedures are performed in Ontario) across 3 hospital corporations (Hamilton Health Sciences [HHS], St. Joseph’s Healthcare Hamilton, and Niagara Health System [NHS]) were nonrandomly allocated to receive at least one of the two planned intervention packages for the subsequent 12 months (ie, the post-intervention period: September 1, 2017 through August 31, 2018; Figure 2). These 6 intervention sites were chosen as a convenience sample based on the investigators’ affiliations. Irrespective of intervention package received (detailed fully in the following sections), all interventions were implemented starting September 1, 2017 at each of these sites. All 6 intervention sites are within the same health care region in southwest Ontario, Canada, which serves approximately 1.4 million people. All other Ontario hospitals will collectively act as a contemporaneous control group [23,24].

### Intervention Packages

#### Groups

The MIPAD study team delivered a multifaceted intervention to reduce morbidity and mortality among post-MI patients through improving long-term adherence to secondary prevention medications. Intervention group 1, comprised of 1 cardiac center and 1 noncardiac hospital (both belonging to the same hospital corporation), received both revised standardized discharge prescription forms and education. Intervention group 2, comprised of the other 4 sites across 2 hospital corporations (including 1 cardiac center), received education alone. The remaining Ontario hospitals, comprising the parallel control group, did not receive any intervention (ie, usual care) [23,24]. The knowledge-to-action framework informed the hypothesis, intervention development, and evaluation of this study [25].

#### Standardized Discharge Prescription Forms

**Figure 3:**
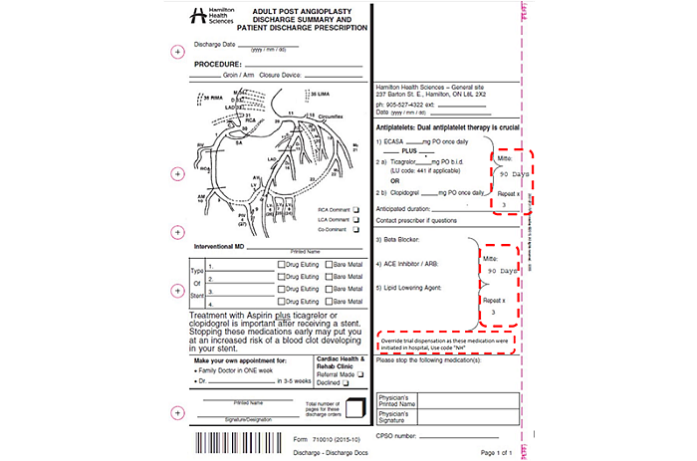
Example discharge prescription from Hamilton Health Sciences with intervention revisions circled.

We implemented a revised standardized discharge prescription form available on all wards where MI patients are managed. The revised prescription form has a default of a 90-day supply with 3 repeats for recommended cardiac medications (ie, primary antiplatelet [aspirin], statin, beta blocker, angiotensin system inhibitor, and secondary antiplatelet). Current discharge prescription forms leave the dispensation amount and repeats to the discretion of the individual physician. To encourage community pharmacists to dispense 90 days’ worth of medications at discharge, in accordance with the revised discharge prescriptions, the Ontario Pharmacists Association recommended that the following statement be included on the discharge prescription: “Override trial dispensation as these medications were initiated in hospital, Use code NH.” This is a standard mechanism to ensure patients with drug coverage from a provincial payer (ie, the Ontario Drug Benefit [ODB] Plan) receive the correct amount of medications on their first fill post-discharge. See Figure 3 for a copy of the discharge prescription form and highlighted revisions. The intervention may be tailored by the physician to allow shorter discharge prescription durations for cost consideration in patients less than 65 years of age and without medication insurance. Monthly monitoring with intervention group 1 ward visits ensured standardized prescription forms were being implemented.

#### Education

The education-based intervention consists of both components detailed in the following sections.

For educational outreach to the hospital staff, educational rounds for all involved health care providers (eg, physicians, residents, nurses) at each of the 6 sites took place at the start of the intervention period to outline the evidence regarding longer prescriptions at discharge and encourage this change in discharge practices. In a preliminary survey of 82 health care providers in the study region, 95% said they would use standardized discharge prescriptions with 3 months of cardiac medication supplies and 3 repeats but less than 50% admitted that they currently provide medication supplies to cover a year (Schwalm, unpublished data). Further education (eg, emails, mailouts, site visits) occurred every 3 months during the intervention period.

For educational support of community pharmacies, in addition to the statement “Override trial dispensation as these medications were initiated in hospital, Use code NH” added to the discharge prescriptions, outreach via personal emails and newsletters to community pharmacies in the study region was undertaken with help from the Ontario Pharmacists Association and Ontario Pharmacy Evidence Network. This outreach was intended to help ensure fidelity of the intervention when medications were dispensed at discharge.

### Data Sources

The following administrative databases held at ICES McMaster will be linked and analyzed for the MIPAD study: (1) CorHealth Cardiac Registry (CCN-CR), which is a clinical database containing information such as demographics, comorbidities (eg, diabetes, hypertension, prior heart disease), and procedure-specific details (eg, referral date, patient wait time, complications, results) for patients who undergo a cardiac procedure (eg, cardiac catheterization, percutaneous coronary intervention, or coronary artery bypass graft surgery) in Ontario [26]; (2) Canadian Institute for Health Information Discharge Abstract Database, which captures demographic, clinical, and administrative information on all hospital inpatient discharges (including deaths, sign-outs, and transfers) in all provinces and territories except Quebec; (3) National Ambulatory Care Reporting System, containing data for all ambulatory care provided in hospital (eg, day surgeries and emergency department visits) or in the community across Canada; (4) ODB database, which captures all prescription medication dispensations for Ontario residents covered under the ODB plan (including those aged 65 years and older); (5) Ontario Health Insurance Plan database, covering physician billings for procedures and consultations; and (6) Registered Persons Database, containing demographic information (including date of death) on all persons registered under the Ontario Health Insurance Plan, which is the majority of Ontario residents. The CCN-CR has been used for numerous quality assurance and research projects, including 1 observational and 1 randomized controlled trial (RCT) conducted by the principal investigators of the MIPAD study [4,27]. Several provinces and territories (including Ontario) also use the Canadian Institute for Health Information Discharge Abstract Database to capture day surgeries [28]. Of the 5 recommended cardiac medication classes post-MI, aspirin (primary antiplatelet) is poorly captured in the ODB, as most dispensations are private pay. Correspondingly, measurement of medication adherence in this study will only concern statins, beta blockers, angiotensin system inhibitors, and secondary antiplatelets.

### Recruitment

**Figure 4:**
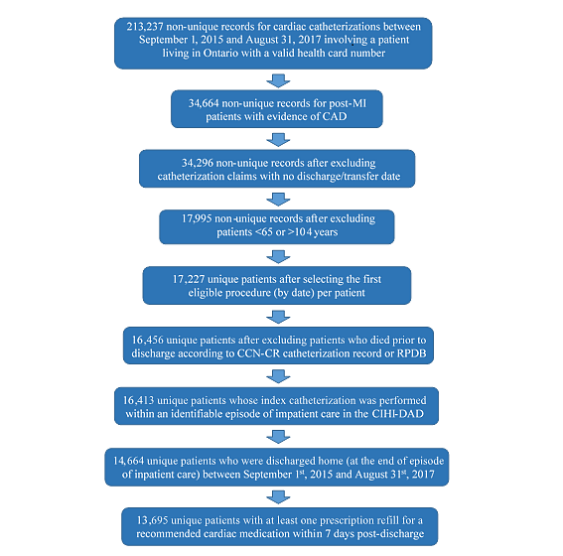
Flow of participants into the study during the pre-intervention period (preliminary data used for power calculation). Notes: Patient age determined at procedure date – 120 days. CAD: coronary artery disease; CCN-CR: Cardiac Care Network – Cardiac Registry; CIHI-DAD: Canadian Institute for Health Information - discharge abstract database; MI: myocardial infarction; RPDB: registered persons database.

Our study population will consist of patients aged 65 years and older who were discharged home from an Ontario hospital between September 1, 2015 and August 31, 2018 following a cardiac catheterization post-MI with evidence of CAD [29]. First, we will identify any CCN-CR claims for a cardiac catheterization with the primary referral reason being a ST-elevation myocardial infarction (STEMI) or non-STEMI with evidence of CAD, defined as left main artery stenosis ≥50% or major epicardial coronary artery stenosis ≥70% (Multimedia Appendix 1) [29]. Patients will be excluded if they meet any of the following criteria: site of procedure missing on their index claim, invalid or missing health card number, non-Ontario resident, <65 years of age (ie, patients who are not automatically eligible for ODB due to age) or ≥105 years of age, or died prior to discharge. Lastly, we will restrict to patients who filled a prescription for at least one medication from one of the 4 recommended cardiac medication classes (ie, statin, beta blocker, angiotensin system inhibitor, or secondary antiplatelet) within 7 days of discharge. This 1-week window was specified based on pre-intervention data, which determined that 93.4% of patients meeting all other exclusion criteria filled at least one recommended cardiac medication within 7 days (with 87.2% having their first fill within 1 day of discharge). We will herein refer to this initial prescription fill as a patient’s index fill. If patients have multiple eligible index cardiac catherization claims within the accrual window — an uncommon occurrence based on pre-intervention data — we only include the patient’s first claim (ie, earliest by date). Figure 4 fully summarizes the application of eligibility criteria to accrue study participants within the pre-intervention period.

### Outcome Measures

#### Primary Outcome

The primary outcome will be long-term cardiac medication adherence assessed at 365 days from discharge. To calculate the primary outcome per patient, first we will identify the number of unique cardiac medication classes (out of 4) — statins, beta blockers, angiotensin system inhibitors, and secondary antiplatelets (ie, prasugrel, clopidogrel, or ticagrelor) — that were dispensed, as per ODB claims on the patient’s index fill date. The PDC will then be calculated per class [30,31], as it is generally a more conservative measure of adherence than the medication possession ratio [30,32]. If patients have 2 or more filled prescriptions for drugs in the same medication class with overlapping days supplied, we will assume the supply was used in sequence, as multiple doses of a drug in the same class would not typically be administered simultaneously [33]. The sum of class-specific PDC values across all classes will then be derived for each patient. Patients with an average PDC ≥80% among those medication classes dispensed at their index fill will be considered as adherent to their cardiac medication regimen [34]. For patients who die during their 1 year of follow-up, their PDC per class will be calculated over the time elapsed (in days) between their index fill and death date. It is not expected that this intervention will increase the number of cardiac medications prescribed to post-MI patients at the time of discharge but rather improve the long-term adherence to the cardiac medications initiated at discharge.

#### Secondary Outcomes

Class-specific adherence (defined as a dichotomous outcome) will also be independently defined as PDC ≥80% for a given drug class at 1 year from discharge. Class-specific medication persistence (defined as a dichotomous outcome) will also be independently defined as having claims for any type of medication within that class from discharge date through the following 365 days with no period of 30 days or more without supply (ie, a discontinuation). The number of unique cardiac medication classes (range, 1-4) filled by a patient at their index fill will also be modelled to investigate whether either intervention meaningfully altered the number of medication classes per patient. Additionally, at the time of a patient’s index fill, we will measure the number of days supplied per medication class and the average number of days supplied across all unique medication classes — both outcomes will be dichotomized (ie, ≥90 days supplied versus <90 days supplied).

In addition to medication adherence–based and persistence-based outcomes, we will assess health care utilization and adverse clinical outcomes at 365 days from discharge including the frequency of outpatient primary care visits, frequency of outpatient cardiology visits, hospitalization for cardiovascular disease, hospitalization due to repeat acute MI, hospitalization due to stroke, repeat cardiac catheterization, coronary revascularization, and death. For both visit-based outcomes, visits occurring on the same day as discharge will be excluded.

### Statistical Analysis

#### Primary Outcome Analysis

Our primary analytical approach will use segmented linear regression analyses of the aggregate monthly data in each group. To promote stability of the time series, we will pool data across the hospitals within each intervention group (ie, standardized discharge prescription forms plus education [intervention group 1] or education only [intervention group 2]). Within each of the 2 intervention groups, we will then independently analyze the aggregated monthly proportions of patients adherent to their cardiac medication regimen at 1 year (expressed as a percentage) using segmented linear regression. In case of nonlinearity of the monthly series, segmented logistic regression analysis of the monthly proportions will be used. Terms will be included for time (in months; treated as continuous [range, 0-35]), intervention (an indicator denoting observations from the post-intervention window), and time post-intervention (defined as time-23 if observation from post-intervention period; 0 otherwise) [35,36]. Serial autocorrelation will be accounted for using first-order autoregressive errors. Visual assessment of model residuals plotted against time will be used to assess goodness of fit. Intervention effects estimated using segmented linear autoregressive error models will be expressed as absolute post-intervention changes in the intercept and slope with corresponding 95% CIs. Post-intervention changes in intercept (or level) and slope (or trend) can respectively be interpreted as the immediate and gradual effects of the intervention on the primary outcome over time [35,36]. Additionally, we will express the overall intervention effect (ie, as combined intercept and slope changes) at the end of the study, on the absolute scale, by comparing the fitted post-intervention trend to the projected secular trend in the final month of observation [35].

The aforementioned segmented regression analyses will compare pre-intervention to post-intervention levels and trends within intervention groups; therefore, groups will act as their own internal controls. Additionally, we will compare these changes to changes observed in the control group (ie, all other hospitals in Ontario). Analysis of the parallel control group — which will be identically specified to the intervention group analyses — will help address threats to internal validity, such as co-occurring interventions or other policy changes that could be rival explanations for any observed changes in our intervention series. In other words, if we detect a post-intervention effect in the parallel control group, it suggests that changes in the underlying patient population or another (unidentified) intervention could have occurred around the same time across Ontario hospitals that might explain away any observed intervention effects.

To account for potential differences in patient case mix over time and among sites, we will conduct additional secondary analyses of the primary outcome with the patient as the unit of analysis. We will use segmented binary logistic regression analysis with terms as specified in previous paragraphs and with the addition of the following patient covariates: age, sex, type of MI (STEMI vs non-STEMI), prior MI, prior cardiac medication use, and site.

#### Secondary Outcome Analysis

Consistent with the secondary analyses of the primary outcome described in the preceding section, all secondary outcomes will be analyzed using patient-level segmented regression analyses with terms for time, intervention, time after intervention, and the following covariates: age, sex, type of MI, prior MI, prior cardiac medication use, and site. The remaining secondary outcomes (time to hospitalization for cardiovascular disease, hospitalization due to repeat acute MI, hospitalization due to stroke, repeat cardiac catheterization, coronary revascularization, and death) will be analyzed at the patient level using Cox proportional hazards regression. Death will be treated as a censoring event for analyses of nonfatal, time-to-event outcomes.

#### Power Calculation

Using the simulation approach developed by Zhang et al [37], we determined that 24 pre-intervention and 12 post-intervention data points (collected at monthly intervals) in each of the 2 intervention groups will achieve 80% power to detect an immediate absolute increase (ie, intercept change) of 10% in the monthly proportion of patients deemed adherent to their recommended cardiac medications using a likelihood ratio test at the 5% level of significance. The anticipated baseline proportion of patients with cardiac medication adherence (ie, average PDC ≥80%) is 75%. The simulation assumed a mean square error for the monthly proportions of 3.1% with 60 patients per month per site, a pre-intervention trend of 0, and an autocorrelation parameter of 0.3.

#### Health Economic Evaluation

We hypothesize that this intervention package is at least cost neutral and potentially demonstrates cost savings when compared to current practice. The cost implications of longer cardiac prescriptions at discharge are expected to be very minimal, with a surplus of medications likely administered in only a minority of cases. We will collect data that will allow us to determine (1) the added costs of medication surplus (ie, wastage) with longer prescriptions in the intervention versus usual care or control periods, (2) the difference in health system costs associated with cardiovascular disease events in the intervention versus usual care or control periods, and (3) the potential for cost savings due to projected reduction in cardiovascular disease events and reduced prescription fees with longer discharge prescriptions.

#### Process Evaluation

Analysis to assess the fidelity of the intervention will be undertaken. Specifically, 50-100 random patient charts from the 3 sites (HHS, St. Joseph’s Healthcare Hamilton, NHS) will be reviewed to assess discharge prescription duration and compare findings against ODB data to assess the level of agreement between the length of the discharge prescription and pharmacy dispensation. In this sample population, we will assess the proportion of patients who received a prolonged duration discharge prescription as reported in hospital charts compared to the proportion who received a prolonged duration discharge prescription in the ODB record. Selection of participant charts for evaluation will be identified from local (HHS, NHS) access to CorHealth registries. These local registries are available for hospital use for approved research and quality assurance work.

## Results

The primary outcome is long-term cardiac medication adherence assessed at 365 days from discharge. We calculated that 24 pre-intervention and 12 post-intervention intervals in each group will achieve 80% power to detect an immediate increase (intercept change) of 10% in the monthly proportion of patients with adherence (ie, proportion of days covered ≥80%) to their cardiac medication regimen at 1 year.

## Discussion

Based on previous observational studies [4,15,16], it is expected that long-term cardiac medication adherence among post-MI patients will improve following the implementation of a standardized discharge prescription length of 90 days with 3 repeats, in conjunction with educational outreach and support, as compared to usual care. Specifically, it is expected that the PDC will improve in each cardiac medication class. The PDC is considered a validated measure of medication adherence [38], and patients with a PDC ≥80% have been shown to have better clinical outcomes [34]. We do not expect that this intervention will increase the number of cardiac medications prescribed to post-MI patients at discharge; rather, we anticipate that standardized discharge prescription forms (in combination with education) will improve long-term adherence to the cardiac medications initiated at discharge.

Previous studies have demonstrated that a 10% increase in cardiac medication adherence in the setting of secondary prevention can result in a 6.7% relative reduction in major adverse cardiac events (MACE), including but not limited to death, MI, stroke, and revascularization procedures [38,39]. Assuming the 1-year MACE rate is 14.7% (based on a very large and high-quality RCT) [40] and estimating that there are approximately 3000 MIs in the study region in 1 year, we would expect the implementation of our intervention to prevent over 30 MACE per year and significantly reduce direct health care expenditures. This estimate only accounts for direct hospitalization costs and does not reflect other costs including rehabilitation, home care, and indirect community care costs. The cost implications of longer cardiac prescriptions at discharge are expected to be very minimal, with a surplus of medications likely administered in a minority of cases.

### Strengths

This study has several strengths. First, revised standardized discharge prescriptions forms are a low-cost and sustainable intervention. If this concept is demonstrated to be effective, the change to prescription duration could be easily implemented into existing discharge policies and even electronic medical records. Second, there is minimal risk associated with this intervention as the 5 discharge cardiac medications are all recommended for a minimum of 1 year post-MI and often lifelong. Cost limitations would be the only foreseeable risk, which are being evaluated as part of the health economic analysis. Finally, the use of administrative databases facilitates a larger study population — including an external control group consisting of all other Ontario hospitals not allocated to an intervention — at lower research operating costs.

### Limitations

While this study proposal highlights the evaluation of a simple, yet novel, approach to improve cardiac medication adherence post-MI, there are methodological limitations. First, the use of administrative databases limits (1) the evaluation of prescription fills rather than swallowing of pills and (2) the assessment of medications and patients not covered by the ODB plan, including aspirin and those <65 years old, respectively. Second, while the interrupted time series analysis can support causal inference, the design is not as robust as an RCT. We do plan to collect appropriate province-wide data throughout this study to inform the design of a future randomized trial. Finally, it is acknowledged that some patients who are classified as nonadherent may have moved away from Ontario, and thus, their prescription claims may be under a different provincial plan for part of the follow-up, leading to potential underestimation of their cardiac medication adherence. The information obtained from this study will help inform the design of a multicenter RCT as an alternative approach to evaluate this proposed intervention.

### Conclusions

In conclusion, the MIPAD study is the first prospective study to evaluate the effects of implementing an institutional policy or standardized discharge cardiac medication prescriptions of maximum duration on long-term medication adherence. The results of this study have the potential to redefine best practices regarding discharge prescribing policy for patients post-MI. Instituting a policy of standardized maximum-duration prescriptions at the time of discharge post-MI is a simple intervention that has the potential to significantly improve long-term medication adherence, thus decreasing cardiac morbidity and mortality. If effective, this low-cost intervention could be easily scaled. Furthermore, since nonadherence is not unique to post-MI patients, this intervention could improve medication adherence in patients with other chronic diseases.

## Appendix

Multimedia Appendix 1 Identification of Cardiac Care Network - Cardiac Registry (CCN-CR) claims for a cardiac catheterization with the primary referral reason being a ST-elevation myocardial infarction (STEMI) or non-STEMI with evidence of coronary artery disease.

## Abbreviations

CAD: coronary artery disease
CCN-CR: Cardiac Care Network - Cardiac Registry
HHS: Hamilton Health Sciences
MACE: major adverse cardiac events
MI: myocardial infarction
MIPAD: Myocardial Infarction Prescription Duration Study
NHS: Niagara Health Services
ODB: Ontario Drug Benefit
OR: odds ratio
PDC: proportion of days covered
RCT: randomized controlled trial
STEMI: ST-elevation myocardial infarction
