# Supplementary material for: Length of Initial Prescription at Hospital Discharge and Long-Term Medication Adherence for Elderly, Post-Myocardial Infarction Patients: Protocol for an Interrupted Time Series Study
Source: JMIR Res Protoc. 2020 Nov 4;9(11):e18981. doi: 10.2196/18981 (PMC7673978; doi:10.2196/18981)
Supplement: Multimedia Appendix 1 [file resprot_v9i11e18981_app1.docx]

The procedure and discharge/transfer dates according to the CCN-CR claims will be denoted; however, while some patients are discharged home directly from the site of the procedure, many are instead admitted or repatriated to another hospital resulting in a discrepancy between the CCN-CR procedure date (and even the recorded discharge/transfer date) and true discharge date. To best accommodate these alternative patient pathways and minimize misclassification of a patient’s discharge date (and corresponding site of discharge), we opted to match each CCN-CR catheterization claim by its recorded discharge/transfer date to CIHI-DAD-based records of an overlapping, uninterrupted episode of inpatient care where the patient was ultimately discharged home on or after the CCN-CR discharge/transfer date. Following the 1:1 match, we then used the end date of the episode of inpatient care as the “true discharge date” (ie, date of index discharge for purposes of study) and correspondingly recorded the hospital from which the patient was discharged.
